# Genome Sequence of Staphylococcus aureus Phage ESa2

**DOI:** 10.1128/mra.00192-23

**Published:** 2023-06-20

**Authors:** Jordan T. Bird, Katie R. Margulieux, Kevin A. Burke, Nino Mzhavia, Richard T. Kevorkian, Damon W. Ellison, Mikeljon P. Nikolich, Andrey A. Filippov

**Affiliations:** a Department of Biochemistry and Molecular Biology, University of Arkansas for Medical Sciences, Little Rock, Arkansas, USA; b Wound Infections Department, Bacterial Diseases Branch, Walter Reed Army Institute of Research, Silver Spring, Maryland, USA; c Bacterial Diseases Branch, Walter Reed Army Institute of Research, Silver Spring, Maryland, USA; University of Rochester School of Medicine and Dentistry

## Abstract

We describe the genome of a lytic phage, ESa2, isolated from environmental water and specific for Staphylococcus aureus. ESa2 belongs to the family *Herelleviridae* and genus *Kayvirus*. Its genome consists of 141,828 bp, with 30.25% GC content, 253 predicted protein-coding sequences, 3 tRNAs, and 10,130-bp-long terminal repeats.

## ANNOUNCEMENT

Staphylococcal phages are effective adjuncts to antibiotics and work against methicillin-resistant Staphylococcus aureus (MRSA) (1). We previously isolated phage ESa1 (2), active against 79% of a 101-strain diversity panel of MRSA isolates, using an efficiency-of-plating assay (3). Here, we describe the genome of phage ESa2, which plates on 58% of MRSA isolates from the same panel and complements ESa1, so they both are potential components of therapeutic phage cocktails.

Phage ESa2 was isolated in 2021 from water of Rock Creek (Montgomery County, MD, GPS coordinates 39°00′57.8″N, 77°05′42.8″W), using S. aureus MRSN 8383. The water sample was filter sterilized and processed as described previously (4). ESa2 was purified by three rounds of single plaque isolation, propagated, concentrated, and filter sterilized (4). Phage DNA was extracted with the QIAamp DNA minikit (Qiagen, Germantown, MD) (4).

The sequencing library was constructed using the KAPA HyperPlus kit (Roche Diagnostics, Indianapolis, IN) and sequenced on an Illumina MiSeq sequencer (Illumina, San Diego, CA) with a 600-cycle MiSeq Reagent kit v3 that produced 300-bp paired-end reads. Default parameters were used for all software unless otherwise specified. Paired-end reads (1,109,178 total) were assessed using FastQC 0.11.9 (5) and trimmed with Trimmomatic 0.39 (6), with the following settings: ILLUMINACLIP, TruSeq3-PE-2.fa:2:30:10; LEADING, 3; TRAILING, 3; SLIDINGWINDOW, 4:24; MINLEN, 60. The ESa2 genome was assembled *de novo* using Unicycler 0.4.8 (7). The genome termini were identified by PhageTerm 1.0.11 (8). Protein coding sequences (CDSs) were annotated using the Pharokka pipeline 1.2.1 (9–19). Amino acid sequence similarity searches were performed using DIAMOND 0.9.14.115 (20, 21).

**FIG 1:**
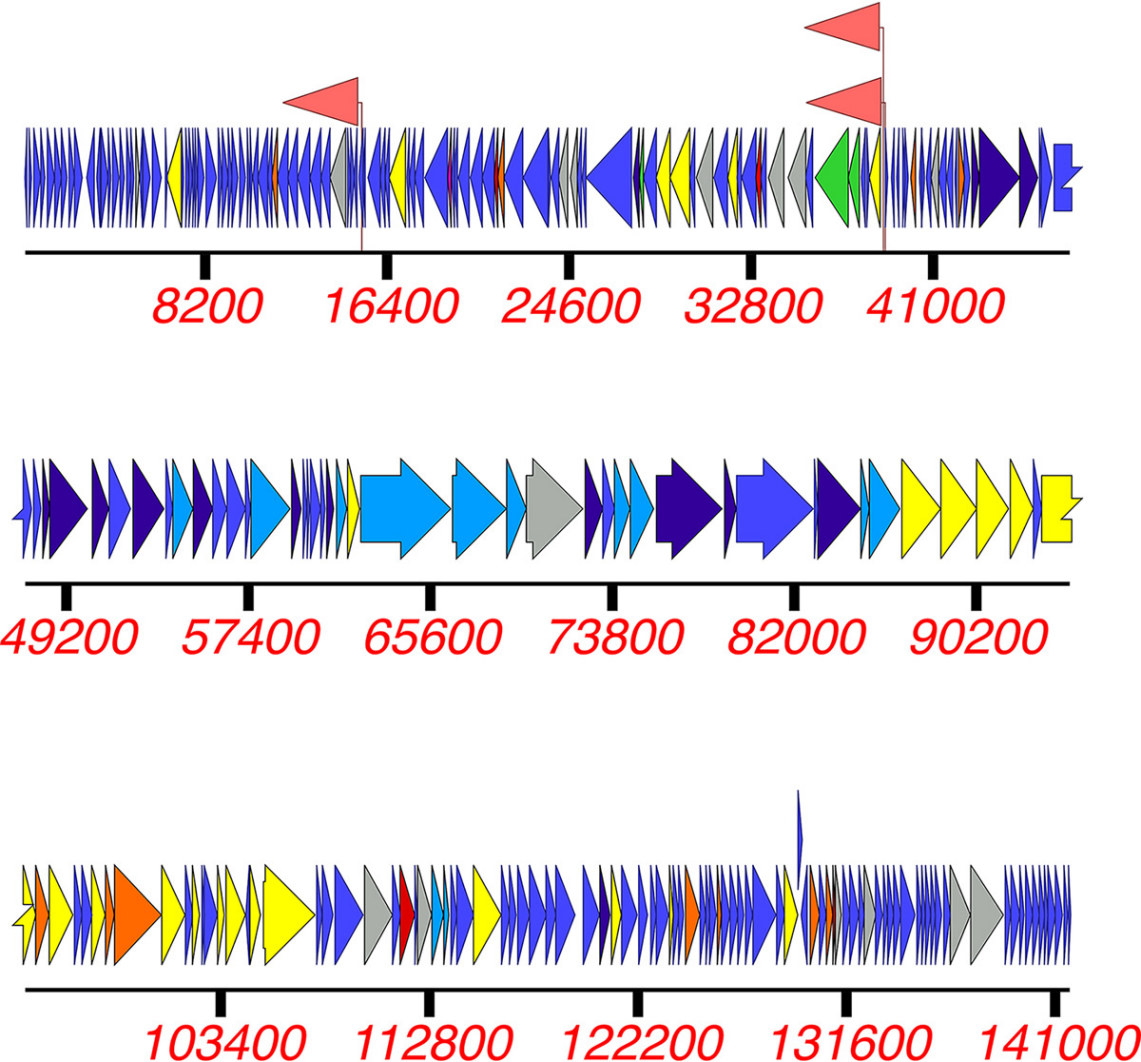
The ESa2 phage genome organization. Colored arrows denote predicted coding sequences, their directions, and functions: unknown (blue), nucleotide metabolism (yellow), moron (orange), integration and excision (pink), transcription regulation (red), head and packing (purple), tail (teal), lysis (green), and other (gray). Pink flags show tRNA sequences.

The ESa2 genome was 141,828 bp long, with a G+C content of 30.25%, and contained 253 predicted CDSs (Fig. 1) and direct terminal repeats of 10,130 bp. Using MASH analysis (19) against the INPHARED database (18), the phage was classified into the family *Herelleviridae*, genus *Kayvirus*. ESa2 showed ~98% MASH sequence identity to 12 other staphylococcal phages, suggesting that all 13 belong to the same species (22). The highest identity (98.3%) was found to GH15 (23, 24) (GenBank accession number JQ686190), HSA30 (GenBank accession number MG557618), and vB_ScoM-PSC1 (GenBank accession number MZ573923). Like phage GH15 (24), ESa2 encodes tRNAs for methionine, phenylalanine, and aspartic acid. GH15 (24), ESa2, HSA30, and vB_ScoM-PSC1 do not have introns and inteins in their genomes that distinguishes them from their relatives.

Although the *Kayvirus* representatives are typically lytic and used as therapeutic phages (25), BACPHLIP (v0.9.6) scored the ESa2 genome at 80%, while the threshold for definitive phage classification as strictly lytic is 95% (26). However, ESa2 putative proteins contained no annotations from Pharokka or DIAMOND to products related to lysogenic lifestyle or gene transfer, including integrases, excisionases, and repressors of the lytic cycle. No hits were detected within the Comprehensive Antibiotic Resistance Database (14) and the Virulence Factor Database (15). Thus, ESa2 appears to be a lytic phage and a candidate for therapeutic use.

### Data availability.

The ESa2 genome BioProject, complete genome sequence, and raw sequence reads were deposited in NCBI, GenBank, and the NCBI Sequence Read Archive under the accession numbers PRJNA933550, OQ428188, and SRR23400555, respectively.
